# Research Progress on the Regulatory Mechanism of the *Waxy* Gene in Rice Starch Synthesis

**DOI:** 10.3390/cimb47090678

**Published:** 2025-08-23

**Authors:** Fei Chen, Yunsheng Song, Yi Jiang, Penghui Cao, Yajie Yu, Minghui Dong, Yulin Xie, Caiyong Yuan, Yongliang Zhu, Zhongying Qiao

**Affiliations:** Crop Research Department, Institute of Agricultural Science in Taihu Lake District, Suzhou 215106, China; feich12345@outlook.com (F.C.); 20173004@jaas.ac.cn (Y.S.); jiangyiedu@163.com (Y.J.); salute272@163.com (P.C.); yuyajien@outlook.com (Y.Y.); mhdong@yzu.edu.cn (M.D.); thsxyl@163.com (Y.X.); hysdycy@163.com (C.Y.); 13145041388@126.com (Y.Z.)

**Keywords:** rice, regulatory metabolism, starch synthesis, *Waxy*, protein structure

## Abstract

Starch serves as a crucial storage substance in both cereal crops and root/tuber crops, with its composition and properties determining the quality of storage organs. The *Waxy* (*Wx*) gene, encoding a key enzyme in starch biosynthesis, plays a pivotal role in this metabolic pathway. However, existing reviews seldom systematically elaborate on *Wx* gene regulatory mechanisms from the perspective of intrinsic molecular networks. Focusing on the model crop rice, this article synthesizes research advances in *Wx*-mediated starch biosynthesis regulation over the past decade. We analyze the structural features of the *Wx* gene and factors influencing its regulatory function during starch synthesis. In conclusion, future research directions are proposed to provide references for *Wx* gene studies in other crops, as well as theoretical foundations for rice varietal improvement and molecular design breeding.

## 1. Introduction

Starch is the main storage form of carbohydrates in cereal crops and crops with tuberous roots and tubers as harvest objects. It provides necessary caloric substances for human survival. Starch biosynthesis is a complex biochemical process [1], which is carried out under the synergistic action of a variety of enzymes [1,2,3]. Research indicates that five key enzymes participate in starch metabolic pathways, including ADP-glucose pyrophosphorylase (ADPGase), granular-bound starch synthase (GBSS), soluble starch synthase (SSS), starch branching enzymes (SBE) and starch debranching enzymes (DBE). Meanwhile, scientists are exploring artificial starch synthesis via chemical methods to address the demand for customized starch products [4].

ADP-glucose, the glucose substrate for starch synthesis, is mainly synthesized by ADP-glucose pyrophosphorylase (AGPase) in the cytoplasm. After ADP-glucose is transported from the cytoplasm to the amyloplast, glucan chains with a certain degree of polymerization are first synthesized, to be used as the precursor primer for starch synthesis as the substrate of plastid starch phosphorylase (Pho1) [5], which initiates the completion of starch synthesis under the action of starch synthase. There are two types of GBSS isozymes, classified into different cotyledons, such as GBSSI found in monocotyledons, and GBSSII mainly found in dicotyledons. The content of GBSSI protein encoded by *Wx* is variant in different species, and is mainly responsible for the synthesis of amylose. GBSSII mainly controls the synthesis of amylose in vegetative organs, such as roots, stems and leaves. There is only a single family of GBSSII in dicotyledons, and its function is similar to the monocotyledons’.

Amylose is usually composed of unbranched short-chain glucans, consisting of α-1, 4-D-glucose units [1]. The formation of amylose is catalyzed by the coordinated action of ADP-glucose pyrophosphorylase (AGPase) and granule-bound starch synthase (abbreviated as GBSS) [6,7]. Amylopectin, the predominant starch component in rice endosperm, consists of α-1,4-linked glucose residues and α-1,6 glycosidic linkages, which are used for the extension of the chain of starch [8,9]. Amylopectin is synthesized by the synergistic catalysis of AGPase, soluble starch synthase (SSS), and starch branching enzyme (SBE, DBE) [10,11]. Specifically, the generation of branching points in the glucan chain is catalyzed by SBE, which is resolved by cutting α-1,4 and α-1,6 glycosidic bonds, thereby enabling and achieving dextran chain extension [12].

The taste quality mainly depends on the content of amylose. The differences of starch content among rice, wheat, sorghum, and potatoes not only affect the cooking and taste quality, but may also affect consumers’ consumption choices. Therefore, it is particularly important to clarify the mechanism of the formation of differences in starch content. In recent years, there have been many research reports on natural mutagenesis, gene editing technology, and RNA interference technology, and favorable *Wx* alleles have been applied in crop quality improvement. However, the synergistic regulatory mechanism of the *Waxy* gene (abbreviated as *Wx*) in starch synthesis and the relationship between *Wx* and other genes in the starch synthesis pathway are not fully understood. Due to the limitation of manuscript length, this article mainly reviews the relevant research on the model crop rice. We have reviewed the latest research progress on the regulatory mechanism of *Wx*, focusing on key genes, promoters, transcription factors, and environmental factors in the starch synthesis pathway. The pleiotropy between genes was also discussed, providing research basis and theoretical reference for the study of other plants.

## 2. Transcriptional Level Differences Affect *Wx* Gene Function

### 2.1. Regulation of Transposons Affects the Function of the Wx Gene

**Figure 1:**
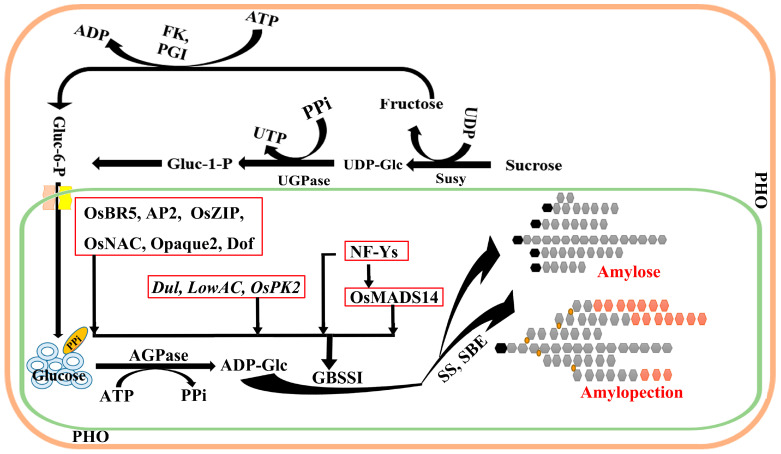
Regulation network of starch synthesis in the endosperm.

In the food processing industry, amylose content directly affects the quality of flour, as it is associated with the starch synthase function encoded by the *Wx* gene, especially affected by nonfunctional *Wx* alleles [13,14]. There are many studies on the quality traits of wheat, which have established their significance in genetics and breeding [15]. It was found that different nonfunctional *Wx* alleles exert different effects on amylose content in wheat flour [15,16,17,18,19]. These ineffective *Wx* alleles often lead to the change of ORF, due to insertion and deletion and the formation of a termination codon, resulting in the early termination of translation [18]. However, interestingly, the study on the evolution of low-amylose rice varieties in tropical areas found that there was a similar 23 bps sequence structure in all tropical glutinous rice. The analysis of bioinformatics retrieval results shows that this sequence seems to verify that tropical glutinous rice varieties may evolve slowly from low-amylose varieties by replicating the *Wx^b^* allele. This implies that the influence of the *Wx* function might depend on the insertion position of these transposable or non-transposable elements, resulting in the loss or change of gene products (Figure 1). Therefore, these findings also provide new ideas and approaches for the developing of germplasm resources in the process of rice variety quality improvement.

### 2.2. Different Binding Sites Affect the Transcriptional Ratio of Wx Gene

The synthesis of amylose is mainly catalyzed by granule-bound starch synthase (GBSS). Phylogenetic analysis and sequence alignment of cereal genome sequences reveal that *Wx* genes are divided into four types: (1) composed of the rice genome; (2) composed of *Wx* genes from millet, maize, sorghum and pea; (3) composed of *Wx* genes from wheat and barley; and (4) composed of *Wx* genes from potato and cassava tubers (Table 1). The *Waxy* gene is a key gene involved in the regulation of amylose synthesis. So far, over eight functional *Wx* alleles in rice have been reported, including *Wx^a^*, *Wx^b^*, *Wx^lv^*, *Wx^in^*, *Wx^op^*, *Wx^mp^*, and *wx*. These alleles can be distinguished by six functional polymorphic loci, namely Int1-1, Ex2-112, Ex4-53, Ex4-77, Ex6-62, and Ex10-115. In order to determine the relationship between these alleles, Zhang et al. used the *Wx* gene sequence alignment method to sequence and analyze a large number of rice germplasm resources. The results indicate that *Wx^lv^* exists in most wild rice varieties [9]. All *Wx* alleles have a cytosine (abbreviated as C) at Ex10-115 and are orthologous with genes encoding GBSSI in other monocotyledonous plants.

An evolutionary tree for the *Wx* allele was constructed using sequences from some cultivated rice varieties. The ancestral *Wx* allele gave rise to the *Wx^lv^* gene, which diversified into distinct haplotypes, such as *Wx^lv^* I, II, III, and IV, through artificial selection. Research confirms that functional locus variations at Int1-1 and Ex6-62 classify *Wx^b^* and *Wx^in^* in *japonica* rice subspecies as *Wx^lv^* I. In contrast, *Wx^a^* and *Wx^op^* underwent base substitutions at Ex10-115 and Ex4-77, categorizing them as *Wx^lv^* II and IV types. *Wx^mp^* and *wx* are derivatives of *Wx^b^*. A 23 bp sequence deletion in the *wx* results in loss of *Wx* function, producing 0% amylose content and a waxy phenotype.

There are two functional alleles with *Wx^a^* and *Wx^b^* in the *Wx* locus [20]. The wild-type allele (*Waxy^a^*, *Wx^a^*) mostly exists in *indica* rice, while the *Wx^b^* gene is widely distributed in *japonica* rice [20,21]. According to the sequence analysis of those, it shows that *Wx^b^* has a guanine (G) to a thymine (T) mutation at the 5′ splicing donor site of the first intron (Table 1). The *Wx* splicing efficiency is led to reduction by this in the process of transcription, and then reducing the amount of GBSSI [22,23,24,25]. The analysis of quantitative trait loci (QTL), based on a genetic mechanism and molecular markers, showed that a wide range of amylose content variation types in rice endosperm were mainly controlled by one major locus (the *Wx* gene) and multiple secondary loci [1,26]. Alterations in GBSSI expression levels or enzymatic activity directly modulate starch composition [25].

Through association analysis, we found that the 5′ terminal splice site of the sequence of the first intron in *Wx^a^*-containing rice varieties was the sequence of AGGTATA. Compared to genotypes harboring the AGTTATA motif, this variety has high levels of *Wx* transcripts and a high content of amylose in the endosperm. Thus, the amylose content in the endosperm is related to the expression level of *Wx* [23]. This conclusion was also verified by the restriction enzyme *Acc* I [27]. In addition, the phenomenon has been found in many rice varieties from Northeast Asia. This suggests that the site with G/T mutation would play an important role in starch synthesis [28]. In the other study, it was found that the sequence of the polymorphic CTn, the microsatellite sequence located in the 5′-untranslated region (5′-UTR) of the first exon in *Wx* locus, may be related to the abundance of amylose content [29]. Therefore, the SNP differences in the encoding regions of different *Wx* alleles may reduce the binding efficiency with starch granules [30,31], and influence the transcriptional expression [32]. *Wx^a^* and *Wx^b^* exhibit transcriptional selective splicing. In addition, the *LowAC1* gene plays an important regulatory role in the transcription process of the *Wx* gene.

This is also an important way to explore low AAC germplasm resources in the process of rice genetic breeding. CRISPR/Cas9 technology was used to edit the promoter, coding region, and non-coding region of the *Wx* gene in the *japonica* rice variety, resulting in rice varieties with different amylose contents. Zeng et al. used *Wx^a^* background material to perform targeted editing on the upstream sequence of approximately 2.0 kb in the promoter region, which includes a 0.9 kb promoter regulatory region and a 1.1 kb 5′-untranslated region (5′-UTR). The first strategy is based on transcriptional regulation, identifying the three cis regulatory elements for editing [12]. The second strategy is to alter intron splicing patterns and affect splicing efficiency by targeting splicing sites including the 5′-UTR of *Wx^a^*. The phenotype identification of mutants revealed that the amylose content of mutants located in the core promoter region was 14.9%, while the amylose content of mutants in the 5′-UTR region, second exon region, and third exon region decreased to below 11.76% and 2%, respectively. The *Wx* gene in ordinary corn can express GBSSI protein normally. However, due to the different jumping vectors of maize transposons within the genome, their effects on the function of *Wx* genes are also different, resulting in changes in starch content in maize grains. These findings collectively highlight the agronomic relevance of amylose content selection, which represents a targeted artificial selection strategy driving crop domestication through starch metabolism optimization. These genetic differences highlight the locus’s pivotal role in *Wx* evolution, supporting molecular design breeding for rice with specific amylose profiles and eating quality characteristics. It creates significant genetic improvements that enhance rice taste quality.

### 2.3. Influence of AGPLs on the Function of the Wx Gene

**Figure 2:**
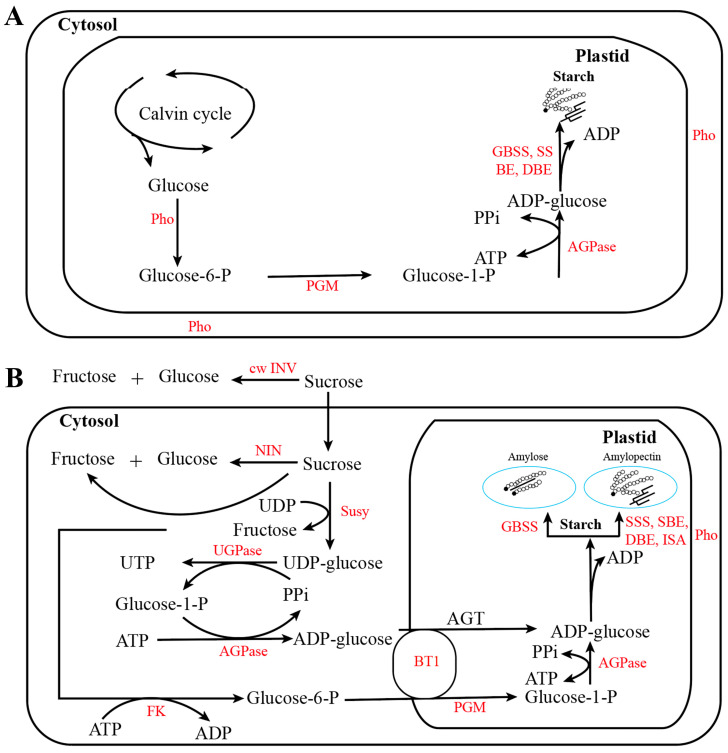
The starch biosynthesis pathway among different organs. The starch biosynthetic pathway in (**A**) photosynthetic tissues and (**B**) the endosperm. Abbreviations for enzymes are as follows: red, corresponding to enzymes included in this article, sucrose synthase, Susy; phosphorylase, Pho; UDP-glucose pyrophosphorylase, UGPase; phosphoglucomutase, PGM; fructokinase, FK; ADP-glucose pyrophosphorylase, AGPase; granule-bound starch synthase, GBSS; starch synthase, SS; starch branching enzyme, BE; debranching enzyme, DBE; isoamylase, ISA; cell wall invertase, cwINV; ADP-glucose transporter, BT1; ADP-glucose transporter, AGT.

Amylopectin is another important component of starch, containing α-1,6 glucosidic bonds synthesized [33]. In contrast to amylopectin, the difference is that amylose has a predominantly linear structure, and the GBSS is involved in the synthesis of amylose (Figure 2). The expression of *AGPL*s is the rate-limiting process of starch biosynthesis [34], a process that is the first key regulatory step [35]. Therefore, AGPase, which provides a substrate for starch biosynthesis, is positively correlated with starch content, indicating that AGPase expression is an important determinant of final starch concentration during fruit ripening [34,36,37,38,39,40].

During the development of grain endosperm, *AGPLs* genes are mainly controlled by the transcription level (Figure 1 and Table 2) [41]. In rice, *AGPLs* are composed of two small subunits and four large subunits [42,43,44,45]. In most plants, ADP-glucose synthesis occurs in the plastids of all tissues. Yu et al. reported that ADP-glucose may also be synthesized outside the plastid, perhaps in the cytoplasm [46]. Conversely, potato varieties overexpressing the plastid adenylate transporter to enhance ADP-Glc synthesis exhibit increased starch production in their tubers compared to the wild type, accompanied by an elevation in the amylose content of the starch. The case of barley appears to suggest that reduced ADP-Glc availability has a more pronounced effect on amylose synthesis than on amylopectin synthesis. However, in barley, maize and rice, there is high expression level of the *AGPLs* gene in different growth stages [43]. Further studies showed that cytoplasmic AGPase accounted for most of the total AGPase activity in endosperm from barley, maize and rice. The phenotypes of maize mutants *shrinken2*, *brittle2* and *brittle1* (*Sh2*, *Bt2* and *Bt1*) show that the synthesis of ADP-glucose in cytoplasm and its introduction into plastids through specific transporters are necessary for starch synthesis to occur at a wild-type rate [47]. The antisense material of the loss-function gene was constructed by RNA interference technology. The comparative analysis results showed that in the process of grain filling, in addition to the decrease of GBSS activity, the activities of AGPLs and SSS in transgenic lines with *GBSSI*-RNAi in the early stage of grain filling were higher than those in the wild type [48,49]. Therefore, the effect of *AGPLs* on the *Wx* gene could be the supply of amylose synthesis substrate, or through interactions with the help of other factors in the starch synthesis network.

### 2.4. Effect of SS on the Functional Regulation of the Wx Gene

The synthesis of starch takes ADP-glucose as the substrate. Whether or not the formation of amylose or amylopectin occurs, the biosynthesis mainly depends on the coordinated action of the key enzymes in the starch synthesis pathway (Table 2). There are four isotypes of starch synthase, which have been proven to be expressed during grain filling [50]. These isoforms are encoded by distinct genes and exhibit tissue-specific expression patterns across developmental stages of the grain. SSI is mainly responsible for the synthesis of the shortest chain, and the synthesis of a longer chain of amylopectin is completed through the further extension of SSII and SSIII [51,52,53], which could change the morphology of starch particles [54,55]. Notably, GBSSI is responsible not only for the synthesis of amylose, but also for the biosynthesis of super-long chains of amylopectin [11,43,55,56,57,58].

The product organs of cereal crops are mainly seeds. The quality characteristics of cereal, the biosynthesis of starch and the distribution of the amylopectin chain are affected by multiple environmental and genetic factors. For example, high temperature (HT) reduces the AC (amylose content) level in the rice endosperm, which is closely related to the decrease of GBSS activity and *Wx* transcript levels [59,60]. It was found that in transgenic plants with *SSI*-RNAi, the starch composition and amylopectin chain distribution changed, but it had little effect on the total starch content [61,62]. This suggests that *SS* deficiency may indirectly affect starch synthesis. That is, the effect of *SSI* deficiency on amylose synthesis and the AC (amylose content) level in rice endosperm may be caused by a significant increase in GBSS activity in grain endosperm [63]. Thus, due to the decrease of GBSS activity under HT, the induced decrease of AC may be partially offset by the opposite effect on AC (amylose content) due to the decrease of *BEIIb* expression. The function of *SSI* in starch biosynthesis exceeds its catalytic activity, and it may participate in the regulation of other starch biosynthetic enzymes with protein–protein interaction or other functions [64]. In addition, different isoforms of *SS* play a synergistic role in the synthesis of amylopectin clusters [65,66,67]. Therefore, these findings imply that the influence of *SS* on the *Wx* gene is likely due to uneven substrate allocation in starch synthesis metabolism.

### 2.5. Transcription Factors Affect the Functional Regulation of the Wx Gene

**Figure 3:**
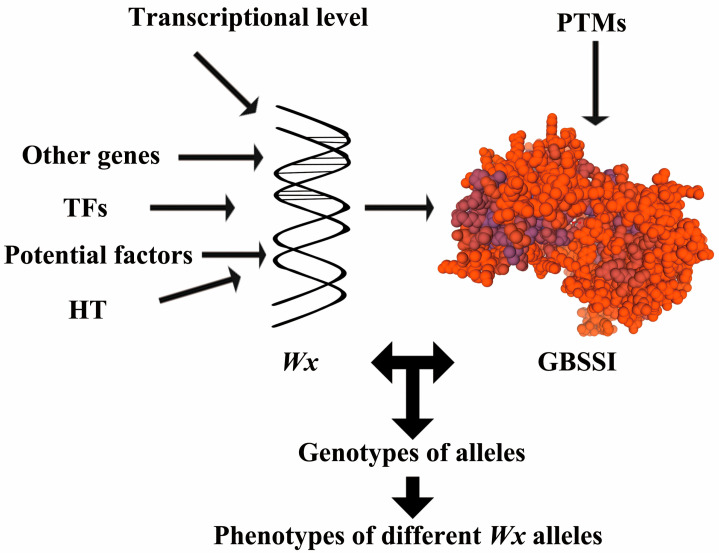
A diagram summarizing the regulatory pathways affecting *Waxy* and GBSSI (3vue.1.A from Swiss-model).

Starch synthesis is a dynamic collaborative regulation process, and the types of key enzymes involved in the synthesis have been relatively clear. Many genes have been reported and identified in many plants (Figure 1 and Figure 3) [3,68], including *AGPLs*, *SS*, *GBSS*, *BE*, *DBE*, *Pho1* (encoded phosphorylase) and PTST (protein targeting to starch) [69]. However, the cooperative interactions among these genes and their regulatory networks remain poorly understood. GBSS overexpression increased amylose content in a *japonica* cultivar rice, due to a polymorphism in the 5′-UTR. The recent overexpression of PTST1 in barley led to only small increases in amylose content (AAC < 10% increase). These results suggested that other factors limit amylose biosynthesis at high GBSS levels. Some studies have shown that the expression of genes is regulated by transcription factors, which affected starch synthesis and accumulation from different plant growth stages. Transcription factors (TFs) play an important regulatory role in the process of plant growth and development. Current research has identified several types of TFs involved in amylose synthesis, namely: (i) the promoter-binding regulation type; (ii) the transcriptional transient expression regulatory type; and (iii) the cooperative type with other genes.

There is no doubt that starch biosynthesis is a complex metabolic pathway. The first category of regulatory factors comprises MYC protein, APETALA2/ethylene response element binding protein and ZIP, which exert stage-specific regulatory roles during plant development. For example, *MYC*-encoded OsBP-5 protein specifically binds to the motif of CAACGTG in the promoter from *Wx* and inhibits its expression, resulting in the reduction of amylose content in mature seeds of transgenic rice [70]. Furthermore, *MYC* interacts with the EREBP protein to jointly regulate the transcription level of *Wx* [71,72,73]. It would increase the promoter activity of starch biosynthesis genes [74], thus breaking the balance of the regulatory network of starch synthesis. Therefore, the identified TF is helpful in exploring the new mechanism of synergistic control of some unknown factors on the synthesis of stored starch in the endosperm of cereal crops [71,75,76,77]. In addition, the negative regulatory factor *NAC* family may belong to the second regular type. The NAC family of TFs, a class of negative regulators, likely represents the second regulatory type. Overexpression of NAC reduces total starch accumulation while upregulating the expression of *AGPL2*, *AGPS2*, *SSI*, *GBSSIIb*, and *BEI* [78]. In the identification of new germplasm resources, a new low-amylose mutant *LowAC1* was isolated and identified. The main characteristics of this effect on AAC was similar to the *Dull* mutant [79], which belonged to the type of transcriptional transient expression regulation. Due to a single nucleotide mutation from G to A, a stop codon was formed in the transcribed sequence by *LowAC1* [80]. This splicing efficiency was affected by this, resulting in the reduction of the protein level of GBSSI encoded by *Wx^b^*.

Transcriptional regulation is a complex process that often plays the role of multiple genes. Gliadin-binding factors PBF, *Dof*, and NF-Ys are categorized into third-type transcription factors [81,82,83,84]. In maize endosperm, the transcription factor *Opaque2* (*O2*) and gliadin-binding factor *PBF* regulate starch biosynthesis. Downregulation of *PBF* and *O2* expression impairs starch synthesis and reduces starch content [81,83]. *Dof* is a nuclear-localized protein with transcriptional activation activity, which can specifically recognize and bind the conserved AAAG motif. Individual members can bind to other target genes, suppress their expression levels, and disrupt starch synthesis, resulting in the production of a defective grain phenotype [85]. It is well known that GBSSI is a key protein responsible for prolonging amylose polymer after recruitment by PTST in the endosperm [86,87], and *GBSSII* is necessary for transient starch biosynthesis in chloroplasts [88,89,90]. NF-Ys are an important transcription factor and play a key role in grain quality control. Combined with RNA-seq data analysis, the NF-YB1 directly binds to the G-box domain of the *Wx* promoter to activate *Wx* transcription, thereby regulating rice quality. Collectively, their approaches or these findings provide a theoretical basis for the genetic improvement of starch crops and a new way for the genetic improvement of crops.

### 2.6. Effects of Other Genes on the Function of Wx

With the continuous improvement of people’s living standards, people’s demand for material culture is also evolving. Starch content is a critical determinant of the eating quality of rice (Figure 3). Two genes encoding GBSS protein, *GBSSI* and *GBSSII*, are responsible for catalyzing the synthesis of amylose. However, there are three mechanisms leading to the change of amylose in cereal crops: (1) Substitution of amino acids. The conversion of acidic amino acid (Asp) to aliphatic (Val), resulting in the inactivation of GBSSI [91]; (2) Base substitution, leading to the premature termination of transcriptional expression. The substitution of cytosine (abbreviated as C) by thymine (shortened T) produces a stop codon and leads to the complete inactivation of *Wx* [92,93]; (3) Structural alterations in gene regions, such as promoter modifications or intron splicing mutations, and so on [94,95].

In the identification of new germplasm resources, some low-amylose mutants were identified. Previous studies have shown that there were specific transfer factors in the endosperm of *japonica* rice. These factors participate in the pre-mRNA alternative splicing in rice and specifically regulate the splicing of genes in the coding process [79,96,97]. In the study of barley, the promoter sequence alterations in *Wx* alleles affect the *Wx* gene expression. Similar results were obtained in previous studies of the waxy barley mutant [98]. However, interestingly, no special regulatory elements were detected in the *seg* mutant, yet its phenotype still exhibited the characteristics of low-amylose content. Further research revealed that reduced *Wx* transcript levels in these *seg* mutants may result from diminished sucrose availability during late endosperm development in the late filling stage [99]. These findings suggest that some other factors, such as the interaction between starch biosynthetic enzymes and protein, and post-transcriptional modification may drive the difference in amylose content variation [100,101].

## 3. The Function Regulation of *Wx* Due to Post-Translational Modification of Protein

Gene expression is temporally regulated, and the manifestation of gene function depends on the synthesis of specific proteins. The structural diversity of proteins determines their functional versatility. The post-translational modification of protein allows organisms to maximize the function of a limited number of genes in organisms at a very low cell cost [102], which reflects this extremely important significance in the study of gene regulation networks. The post-translational modification (PTM) is a covalent modification process. PTM diversifies protein function through sequence-specific modifications, such as acylation, phosphorylation, glycosylation, and so on, in which the main protein structure is changed in a sequence-specific manner, and finally the function is diversified [46].

The main types of modification of PTMs primarily include glycosylation, acetylation, phosphorylation, ubiquitination, methylation, and nitration [103]. Among these, phosphorylation stands out as one of the most biologically significant PTMs in molecular biology research [104]. In the study of molecular biology, phosphorylation was the most commonly used form of PTM (Figure 1 and Figure 3). In particular, the most critical PTMs detected in plants include His, Tyr, Ser, Asp, Thr and other amino acids prone to phosphorylation. However, this rarely covalently binds to hydroxyproline in protein phosphorylation [105,106,107]. Protein phosphorylation has become one of the main PTMs, and plays a crucial role in multiple aspects. Interestingly, protein phosphorylation is currently the only confirmed form of PTM [108]. Using this, the study of coding phosphorylation characteristics will contribute to better molecular design, breeding and targeted improvement of rice quality [109].

The identification of phosphorylation sites in GBSSI was convenient to reveal more about the PTM of GBSS, which may lead to the discovery of new mechanisms affecting amylose content [110,111]. Previous studies have found that a decrease in the phosphorylation of *Wx* at Ser^34^ affects the activity of GBSSI. Liu et al. reported that the substitution of Asp^165^ with Gly^165^ had no significant effect on the activity of GBSSI in vitro [31], but this mutation significantly modifications appear to reduce the binding ability equally between GBSSI and starch particles. Huang et al. and Zeng et al. reported that in *Wx* loci, protein stability, phosphorylation, ADP-glucose binding and other sites controlling the function of GBSSI may be the targets of PTM modification or translation [112,113]. Conserved phosphorylation sites of GBSSI played an important role in starch biosynthesis [110]. Further, it was only the phosphorylation level of Ser^415^ that changed, which led to the change of amylose content. Additionally, three GBSSI-conserved phosphorylation sites of GBSSI were detected by Chen et al. [114]. The expression level of *Wx* was directly affected by PTMs, which led to decreased amylose contents [31]. Therefore, the change in specific gene-conserved phosphorylation sites in the starch synthesis pathway could regulate the content of starch biosynthesis in crops [109,115]. Studies have observed Lysine acetylation, lysine succinylation, and DNA methylation in crucial enzymes of the starch pathway [116,117,118]. The modifications of these PTMs seem to indicate that they can balance the relationships within metabolic networks and adjust the turnover rates of carbon and nitrogen to meet the needs of different growth stages through modifying multiple proteins.

## 4. Additional Factors Affect the Functional Regulation of *Wx*

Higher ambient temperature significantly reduces the amylose content in the endosperm, which may result from decreased enzymatic activity and/or temperature-dependent regulation of starch in grain filling [119,120]. Therefore, the starch content and nutritional quality in gramineous plants are not only genetically controlled but also significantly compromised by extremely high-temperature events, especially during grain filling [121]. Recently, studies have shown that high temperature (HT) suppresses amylose synthesis by impairing GBSSI activity in the endosperm and disrupts synergistic interactions within the starch synthesis pathway [122], ultimately resulting in a series of phenotypes such as seed shrinkage, powder and no endosperm [59,123]. With the intensification of global climate change, and the prolonged duration of extreme weather, the impact on rice quality and safe food production is also escalating. This underscores the urgent need to identify heat-tolerant germplasm resources and elucidate molecular adaptation mechanisms.

Recently, it was found that different *Wx* alleles were not sensitive to sucrose at different sucrose concentrations by in vitro incubation (studies using in vitro incubation assays revealed that the enzymatic activity of various *Wx* alleles remains unresponsive to sucrose across a broad concentration gradient) [43,124,125]. With increasing ABA concentration, the inhibition of this synthase is greater [126]. ABA treatment in seed development not only inhibited the transcription of GBSSI, but also inhibited the transcription of GBSSII in the middle stage of seed development. Despite these findings, little is known about the molecular mechanism of ABA in controlling the entry of sucrose into the endosperm and its involvement in regulating starch metabolism during grain filling [127]. Thus, the signaling network governing endosperm starch synthesis under abiotic stress is incompletely mapped, necessitating targeted investigations to bridge this knowledge gap. Elucidating these mechanisms will advance precision cultivation strategies to optimize rice yield and quality under fluctuating environmental conditions.

## 5. Conclusions and Prospects

The industrial significance and extensive application potential of starch provide substantial research material for investigations across various crops. The *Wx* gene is one of the most widely studied genes in starch metabolism research. Through molecular biology techniques, analysis of starch biosynthesis is an important pathway for cultivating crops with specific starch content. Klosgen et al. determined the genome and cDNA sequence of the wild-type *Wx* in maize. Due to transposition effects in the maize genome, the *Wx* gene forms multiple types of mutations [128]. This provides a genetic basis for the formation of varieties with different amylose contents (Table 1). The identification of amylose content with different *Wx* alleles in rice showed a continuous variation in amylose content, suggesting functional differences in *Wx* among different alleles.

In other cereal crops, the presence of multiple chromosome sets and gene copy variations often leads to phenotypic diversity among individuals sharing identical genes. Extensive research has been documented regarding *Wx* gene studies in wheat crops [129,130]. The *Wx*-B1a gene is the main gene for wheat amylose synthesis (21–22%), with alleles *Wx*-D1a and *Wx*-A1a producing 20–21% and 15–18% amylose, respectively. *Wx*-A1, *Wx*-B1, and *Wx*-D1 exhibit non-functional characteristics [129,130,131]. Santosh et al. demonstrated that the viscosity of tetraploid miliaceum (*P*. *miliaceum* L.) is regulated by the *Wx*-2 and *Wx*-38 loci of the *Wx*. At the same time, the screening and identification results of local varieties of millet also confirmed these functional loci [132,133]. Therefore, the *Wx* gene plays an important regulatory role in the changes of starch content during starch synthesis.

Rice quality is determined by coordinated glucose metabolism processes including carbohydrate, protein metabolism and fat metabolism. The quality of rice mainly includes milling quality, appearance quality, and cooking and eating quality. With the development of the times and the continuous improvement of people’s material living standards, food safety and healthy diet have also become critical determinants of agricultural research priorities. The cooking and eating quality has become the focus of attention. Higher and higher requirements are put forward for superior quality traits of crops. It is very important to strengthen research on the molecular biology, biochemistry and enzymatic mechanism of the formation. In conclusion, *Wx* is one of the key genes during starch synthesis, playing an important role in quality. Notwithstanding, great achievements have been made in the study of the *Wx* gene structure, its function, and the genetic effect of polymorphism. However, the molecular mechanism of its regulation in the starch synthesis network is still unclear, and the following problems still exist: (1) The effect of *Wx* from multiple perspectives needs to be studied, and more variation types need to be explored; (2) The regulatory and/or fine-tuning mechanism of *Wx* and other genes in starch synthesis is not yet clear. Specifically, what is the molecular mechanism for identifying the early leading short-chain in starch synthesis? Additionally, how is the *Wx* gene regulated in dicotyledonous crops such as pea, soybean, and rapeseed? In addition to accelerating the excavation and identification of different germplasm resources, it is also urgently necessary to transform specific genes and create new varieties of resources. Therefore, the future research thrusts of *Wx* should start from the following aspects: (1) The new germplasm resources are identified to enrich the regulation mechanism of amylose; (2) Although two GBSS isoforms exist in rice, their functional interactions within the starch synthesis regulatory network remain poorly characterized; (3) Multi-omics integration is required to elucidate the coordinative roles of *Wx* in starch metabolism; (4) The coordination mechanism between *Wx* and other genes should be explored from different omics perspectives, laying the foundation for further improving and utilizing the quality of rice through this gene.

## Figures and Tables

**Table 1:** Genotypes and phenotypes of different *Wx* alleles in different crops.

Crop	Allele Gene	Genetic Variation Loci in *Wx* Gene	Endosperm Phenotype
Rice	*Wx^a^*	G at Int1–1 and T at Exon-10	High amylose content
	*Wx^b^*	T at Int1–1 and C at Exon-10	Low amylose content
	*Wx^lv^*	G at Int1–1 and C at Exon-10	High amylose content
	*Wx^in^*	A single nucleotide polymorphism in exon-6	Intermediate-type
	*Wx^mw/la^*	A single A to C substitution on exon-6	Low AC
	*Wx^op/hp^*	G in intron-1	Very low AC
	*Wx^in^*	A single nucleotide polymorphism in exon-6	Intermediate-type
	*Wx^lv^*	G at Int1–1 and C at Exon10	High amylose content
	*Wx^op/hp^*	G in intron-1	Very low AC
	*Wx^mp^*	T in Int1–1 and A at Exon4–53	Very low AC
	*wx*	23 bp duplication inserted in exon-2	Very low or no AC
Maize	*wx-m1*	409 bp insertion in exon-9	–
	*wx-B2*	128 bp insertion in Exon-11	Waxy
	*wx-C34*	Deletion	Waxy
Wheat	*Wx-A1*	Wild type	Non-waxy
	*Wx-B1b*	Complete deletion	Waxy
	*Wx-A1b*	Deletion of 23 bp in the second exon–intron junction	Waxy
Sorghum	*wxa*	4 kb large insertion in exon-3	Waxy
	*wxb*	Missense mutation	Waxy
Cassava	*Wx*	A single base substitution in exon-11	Non-waxy
	*wx*	A single base deletion in exon-6	Waxy
Barley	*Wx-CDC*	397 bp deletion and a 193 bp insertion	Waxy
	*Wx-Bowman*	11 bp insertion in the promoter region	Non-waxy
Foxtail millet	*Type I*	Wild type	Non-waxy
	*Type II (TSI-1)*	343 bp insertion in intron-1	Non-waxy
	*Type III (TSI-6)*	4050 bp insertion in intron-1	Low-amylose
Barnyard millet	*EeWx1*	Wild type	Non-waxy
	*EeWx2*	Wild type	Non-waxy
Proso millet	*WxL_C_*	Wild type	Non-waxy
	*WxS_−15_*	15-bp deletion in exon-10	Waxy
Job’s tears	*EeWx*	275-bp deletion in exons 10–11	Waxy
Amaranths	*Type Ia*	A single nucleotide polymorphism on exons-1 and -6	Non-waxy
	*Type IIIa*	A single nucleotide polymorphism on exons-1 and -6	Waxy

**Table 2:** Enzymes involved in starch synthesis in the filling of cereal endosperm.

Enzymes	Rice (*Oryza sativa* L.) Gene Symbols	Wheat (*Triticum aestivum* L.) Gene Symbols	Maize (*Zea mays* L.) Gene Symbols
ADPG pyrophosphorylase (AGPase, EC 2.7.7.27)	*OsAGPL1*	*TaAGPL1*	*ZmAGPL1*
	*OsAGPL2*	*TaAGPL2*	*ZmAGPL2*
	*OsAGPL3*	/	*ZmAGPL3*
	*OsAGPL4*	/	*ZmAGPL4*
	*OsAGPS1*	*TaAGPS1*	*ZmAGPS1*
	*OsAGPS2 a/b*	*TaAGPS2 a/b*	*ZmAGPS2 a/b*
Granule-bound starch synthase (GBSS, EC 2.4.1.21)	*OsGBSSI*	*TaGBSSI*	*ZmGBSSI*
	*OsGBSSII a/b*	*TaGBSSII*	*ZmGBSSII a/b*
Soluble starch synthase (SS, EC 2.4.1.21)	*OsSSI*	*TaSSI*	*ZmSSI*
	*OsSSII a/b/c*	*TaSSII a/b*	*ZmSSII a/b/c*
	*OsSSIII a/b*	*TaSSIII a/b*	*ZmSSIII a/b*
	*OsSSIV a/b*	/	*ZmSSIV*
Starch branching enzyme (SBE, EC 2.4.1.18)	*OsSBEI*	*TaSBEI*	*ZmSBEI*
	*OsSBEII a/b*	*TaBEII a/b*	*ZmBEII a/b*
	*OsSBEIII*	*TaBEIII*	*ZmBEIII*
Starch/a-glucan phosphorylase (PHO, EC 2.4.1.1)	*OsPHOL*	*TaPHOH*	*ZmPHOH*
Protein targeting to starch (PTST)	*OsGBP*	*TaBGC1*	*GPM177*

## Abbreviations

The following abbreviations are used in this manuscript.

AGPase	ADP-glucose pyrophosphorylase
PHO	Phosphorylases
PTST	Protein targeting to starch
GBSS	Granule-bound starch synthase
SS	Starch synthase
SBE	Starch branching enzyme
Susy	Sucrose synthase
UDPG	Uridine diphosphate glucose
AAC	Apparent amylose content
ADPG	ADP-glucose
RNAi	RNA interference
